# Impact of surgical intervention trials on healthcare: A systematic review of assessment methods, healthcare outcomes, and determinants

**DOI:** 10.1371/journal.pone.0233318

**Published:** 2020-05-22

**Authors:** Juliëtte J. C. M. van Munster, Amir H. Zamanipoor Najafabadi, Nick P. de Boer, Wilco C. Peul, Wilbert B. van den Hout, Peter Paul G. van Benthem

**Affiliations:** 1 Department of Otorhinolaryngology and Head and Neck Surgery, Leiden University Medical Center (LUMC), Leiden University, Leiden, the Netherlands; 2 Leiden University Neurosurgical Center Holland (UNCH), LUMC and The Hague Medical Center (HMC), Leiden, the Netherlands; 3 Department of Biomedical Data Science–Medical Decision Making, Leiden University Medical Center, Leiden University, Leiden, the Netherlands; Zagazig University, EGYPT

## Abstract

**Background:**

Frameworks used in research impact evaluation studies vary widely and it remains unclear which methods are most appropriate for evaluating research impact in the field of surgical research. Therefore, we aimed to identify and review the methods used to assess the impact of surgical intervention trials on healthcare and to identify determinants for surgical impact.

**Methods:**

We searched journal databases up to March 10, 2020 for papers assessing the impact of surgical effectiveness trials on healthcare. Two researchers independently screened the papers for eligibility and performed a Risk of Bias assessment. Characteristics of both impact papers and trial papers were summarized. Univariate analyses were performed to identify determinants for finding research impact, which was defined as a change in healthcare practice.

**Results:**

Sixty-one impact assessments were performed in 37 included impact papers. Some surgical trial papers were evaluated in more than one impact paper, which provides a total of 38 evaluated trial papers. Most impact papers were published after 2010 (n = 29). Medical records (n = 10), administrative databases (n = 22), and physician’s opinion through surveys (n = 5) were used for data collection. Those data were analyzed purely descriptively (n = 3), comparing data before and after publication (n = 29), or through time series analyses (n = 5). Significant healthcare impact was observed 49 times and more often in more recent publications. Having impact was positively associated with using medical records or administrative databases (ref.: surveys), a longer timeframe for impact evaluation and more months between the publication of the trial paper and the impact paper, data collection in North America (ref.: Europe), no economic evaluation of the intervention, finding no significant difference in surgical outcomes, and suggesting de-implementation in the original trial paper.

**Conclusions and implications:**

Research impact evaluation receives growing interest, but still a small number of impact papers per year was identified. The analysis showed that characteristics of both surgical trial papers and impact papers were associated with finding research impact. We advise to collect data from either medical records or administrative databases, with an evaluation time frame of at least 4 years since trial publication.

## Introduction

Research impact is defined as an effect on, change or benefit to the economy, society, culture, public policy or services, health, environment or quality of life[1–7]. Despite the introduction of multiple research impact evaluation frameworks by governments and funding bodies (e.g. the Research Excellence Framework and the Payback framework[8]), the actual methods used in case studies vary widely, and therefore it remains unclear which methods are most appropriate for evaluating research impact in different fields of healthcare research.

In the field of surgical research, the translational impact of surgical trials on clinical practice is rarely evaluated, hampering optimal implementation and de-implementation of surgical interventions[9]. It was suggested that reducing low-value surgical interventions, based on high-quality evidence, can save €153 million per year in the United Kingdom alone[10–14]. High-quality surgical research has increased worldwide in the past decades[15]. But to actually reduce the use of these low-value interventions, high quality research evaluating the impact of clinical trials is warranted as well, measuring relevant and actable outcomes on healthcare[5, 8, 16]. This statement is supported in The Innovation, Development, Exploration, Assessment, and Long-term study (IDEAL) Framework, which was introduced to improve quantity and quality of surgical research[9, 17]. For example, Ainsworth et al. showed that the overall impact of a trial on the effectiveness of axillary lymph node clearance did not significantly change practice, although the trial had important implications for clinical practice. It was recommended to better inform patients of their treatment options as a result of the outcomes from the impact trial[18].

A standardized approach of research impact evaluation could address methodological discrepancies and better inform decision makers and healthcare practitioners[5, 7, 19–23]. Therefore, the aim of this systematic review was to identify and review the methods used to assess the impact of surgical intervention trials on healthcare in case studies to provide a strategy for surgical research evaluation to researchers, healthcare practitioners and decision makers. In addition, we assessed possible determinants for finding surgical impact in terms of characteristics of the original trial and characteristics of the impact study.

## Methods

This systematic review was reported according to the Preferred reporting items for systematic review and meta-analysis protocols (PRISMA) guidelines and was registered in the PROSPERO register (registration number: CDR42018106812) before title-abstract screening and full-text screening was performed[24].

### Literature search and eligibility criteria

PubMed, Embase, Web of Science, and The Cochrane Library were searched systematically on March 10, 2020. Together with a trained librarian we compiled our search strategy for impact papers consisting of four concepts: “surgery”, “clinical trials”, “impact”, and “clinical practice”. The full search strategy can be found in the Appendix. We included papers that investigate the impact of surgical intervention trials as defined in the Research Excellence Framework[1]: “Research impact was defined as an effect on, change or benefit to the economy, society, culture, public policy or services, health, environment or quality of life, beyond academia”. Papers were excluded when they investigated the impact of non-surgical trials, the impact of surgical treatments on healthcare not related to trial publication, or the impact of future research or guideline implementation without the impact of the actual surgical trial on healthcare. Also, (descriptions of) original investigations, study protocols, expert opinions, letters to the editor, (economic) analyses of interventions, and papers describing methodological implications for impacts studies were excluded. Screening of eligible articles was performed independently by two authors (JM and NB). If agreement could not be reached between the two authors, the opinion of two other authors (WH and AZ) was requested to reach consensus. For each impact paper, the associated trial paper (or papers) was (were) identified from the provided references. We also searched databases on most important research impact frameworks mentioned in previous reviews[5, 7, 19–21, 23], but did not discovered additional impact papers.

### Risk of bias assessment

Two authors (JM and AZ) independently assessed the Risk of Bias (RoB) of both the articles describing the surgical trials (trial papers) and the impact papers. For the trial papers, quality was calculated using the Methodological Items for Non-Randomized Studies (MINORS), which includes important quality assessments applicable to randomized studies as well[25]. The ideal score is 24 points for comparative studies, and 16 points for non-comparative studies. No tool exists to specifically assess the quality of studies estimating the impact of trials on healthcare practice. We used the Robins-I tool to assess Risk of Bias since we feel this fits best to analyze impact assessments[26].

### Data extraction

Data were extracted from each impact paper and each trial paper by two authors (JM and NB). From the impact papers, we extracted the following data: primary author, publication date, surgical specialty, region of data collection and data collection methods, timeframe of evaluation in years, outcome measurement, number of time points for outcome measurement, analysis methods, limitations, and main results. Conclusions as reported by the authors of the impact papers were divided in two groups: yes (research impact occurred) and no (no research impact or no clear statements made by the authors). From the trial papers, we extracted the following data: publication date, type of comparison (surgery vs. surgery, surgery vs. watchful waiting, or surgery vs. non-surgical treatment), implementation vs. de-implementation, sample size, economic evaluation performed (possibly in a separate paper), study design, external funding, and conclusion made by the authors.

### Analyses

Univariate analyses were performed to identify determinants of both the trial papers and the impact papers on finding research impact. Conclusions made by the authors of the impact papers were used to define whether impact papers did or did not found research impact. The following characteristics of the trial papers were analyzed: time since publication trial paper in months, economic evaluation performed (yes vs. no), type of comparison (surgery vs. surgery, surgery vs. watchful waiting, and surgery vs. non-surgical treatment), implementation versus de-implementation, specialty (oncological surgery as a subspecialty of general surgery versus other specialties (e.g. non-oncological general surgery, neurosurgery, trauma surgery), external funding (yes versus no), sample size, RoB score (MINORS), and whether a significant difference was found for the treatment outcomes (yes versus no). For the impact papers we examined: design (purely descriptive, comparative analysis, or time series analysis), data collection (opinion of physicians, medical records, administrative databases), case-mix presentation (yes versus no), the continent where the evaluation was performed (North America versus Europe), timeframe of evaluation (range between years that were evaluated), months between publication impact paper and trial paper, months between literature search and impact paper, and RoB score (Robins-I). For continuous variables, we performed an independent *t-*test or Mann-Whitney U test in case of non-parametric data, and Chi-square tests for categorical variables or Fisher exact tests in case of less than five observed values per category, all two-sided with a statistical significance level of *P*<0.05. Post-hoc analyses were performed for significant findings for possible determinants with more than two groups, using Fisher exact tests for all possible comparisons between groups, with a Bonferroni correction for multiple testing. SPSS Statistics software (version 26; IBM Corp) was used for all statistical analyses.

## Results

### Search strategy and selection

**Fig 1:**
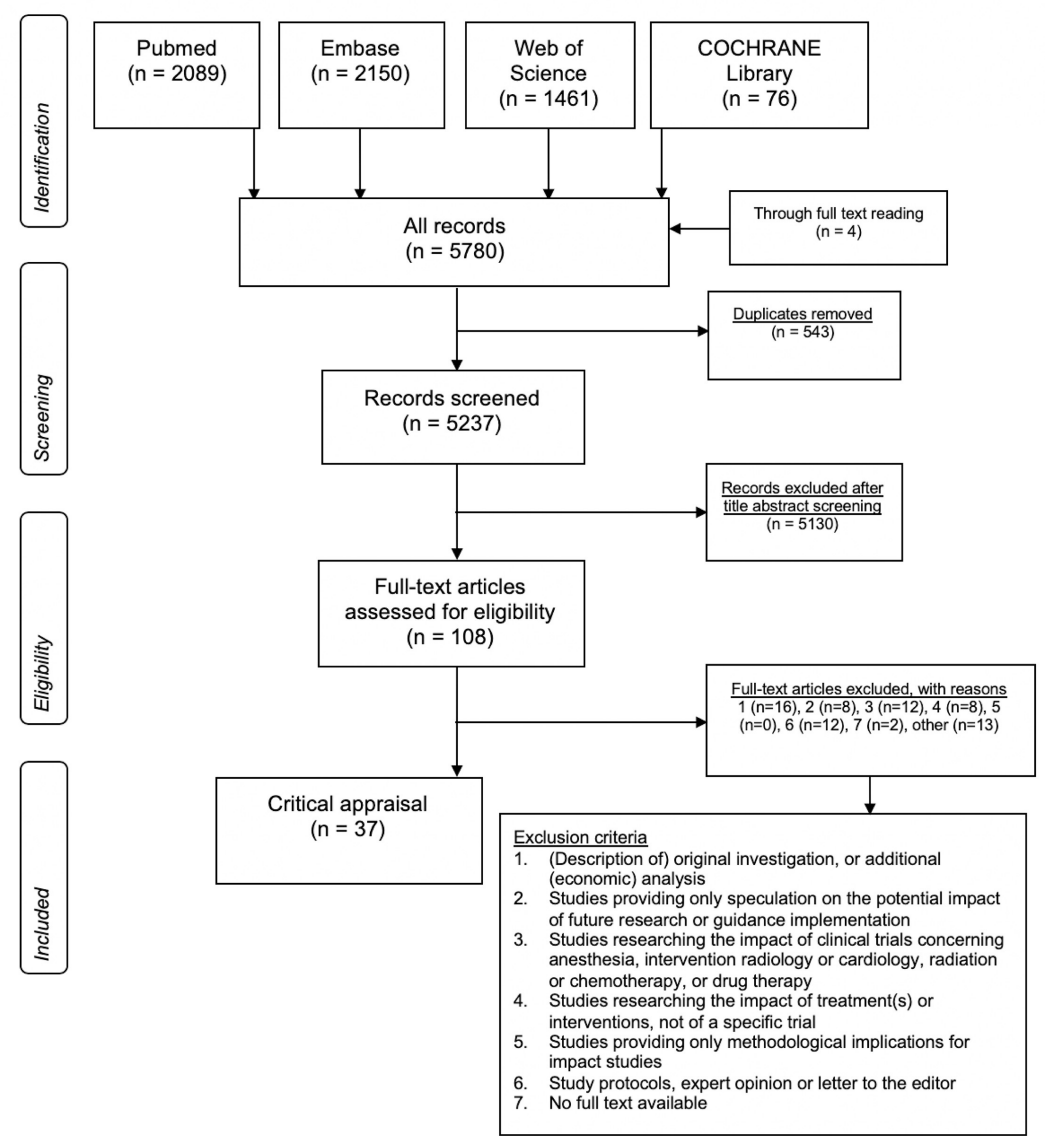
PRISMA flow diagram for study selection.

The search identified 5237 unique publications, of which 108 full-text articles were evaluated for eligibility and 37 included in the analysis. Reasons for exclusion are presented in the Flow Diagram (Fig 1).

### Characteristics and quality assessment of the impact papers

**Fig 2:**
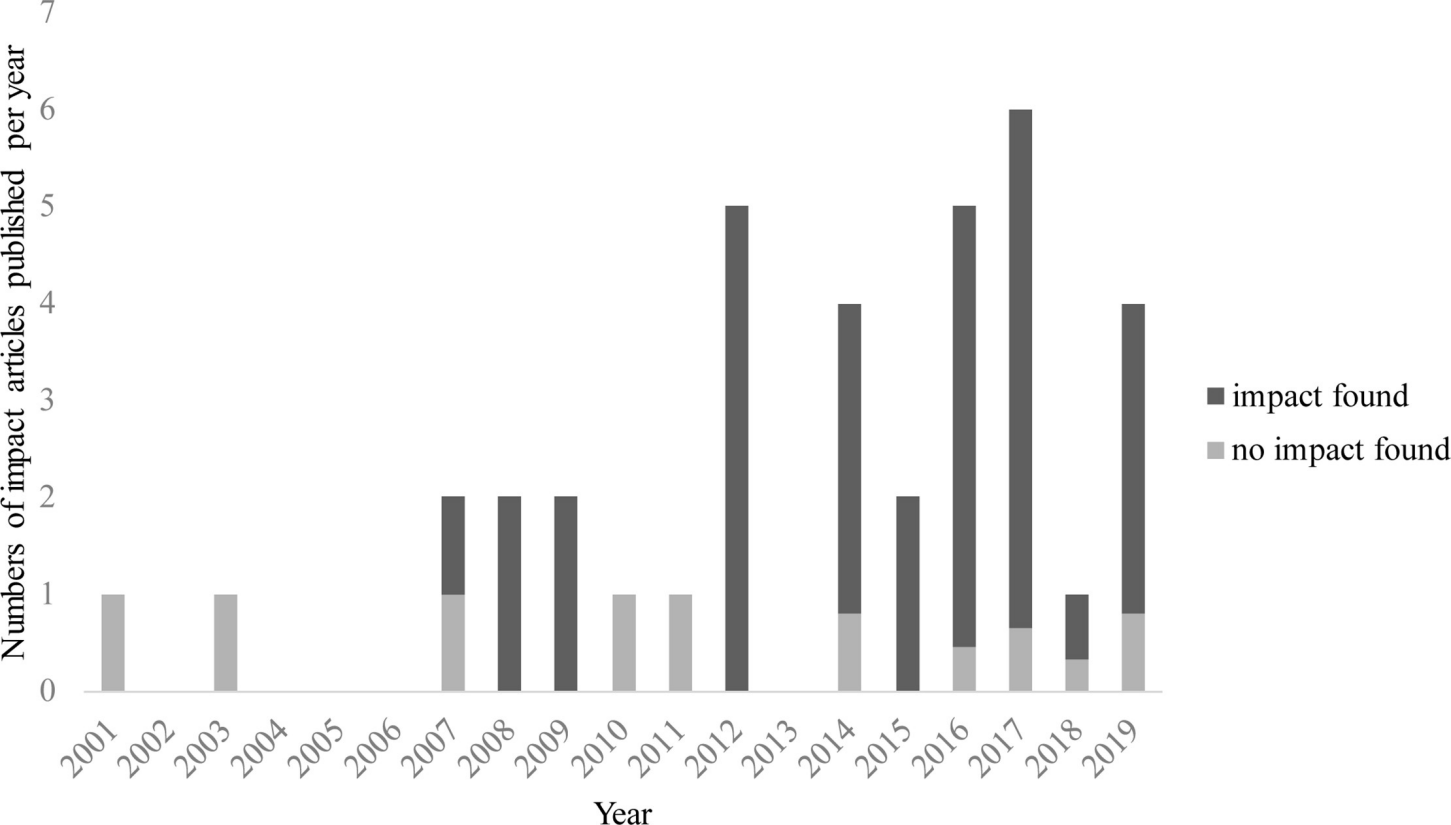
Number of published impact papers per year.

The number of papers increased over time, with a maximum of 6 surgical intervention trial impact papers per year issued in 2017[27–32] (Fig 2).

Surprisingly, none of the included impact papers mentioned the use of a methodological framework to assess the impact of the trial papers. Most impact papers were published in the surgical oncology field[28, 33–43] or neurosurgical field[27, 30, 44–50] (Table 1, details in S2 Table) and were conducted in North America[27, 30–32, 34–40, 43, 44, 46, 47, 49–57], less often in Europe[33, 41, 42, 45, 48, 58–63], and not on other continents.

**Table 1:** Characteristics of the surgical impact papers.

Characteristics	Impact papers (no. = 37)
Specialty, no. (%)	
*General surgery*	2 (5)
*General surgical oncology*	12 (32)
*Trauma surgery or orthopedic surgery*	7 (19)
*Vascular surgery*	5 (14)
*Otorhinolaryngology*	2 (5)
*Neurosurgery*	9 (24)
Publication period, no. (%)	
*2000–2004*	2 (5)
*2005–2009*	6 (16)
*2010–2014*	11 (30)
*2015–2019*	18 (49)
Data collection, no. (%)	
*Medical records/hospital data*	10 (27)
*National*, *administrative database*	22 (59)
*Opinion of professionals (questionnaire study)*	5 (14)
Study design, no. (%)	
*Descriptive only*	3 (8)
*Pre-post analysis (comparing two time periods)*	9 (24)
*Trend analysis over consecutive years*	11 (30)
*Pre-post analysis and trend analysis combined*	9 (24)
*Interrupted time series analysis or spline regression analysis*	5 (14)
Case-mix presented, no. (%)	
*Socio-economic information*	2 (5)
*Disease characteristics*	3 (8)
*Both*	14 (38)
*None*	18 (49)
Costs evaluation, no. (%), yes	5 (14)
Timeframe, no. (%)	
*After*	3 (9.1)
*Before–after*	30 (90.9)
Years evaluated before trial, median (IQR)	4.0 (2–8)
Years evaluated after trial, median (IQR)	3.0 (2–6)
Time interval between points^a^, no. (%)	
*Quarters*	3 (9)
*Half year*	1 (3)
*Years*	29 (88)
Impact^b^^,^^c^, no. (%), yes	49 (79)

^a^ 33 papers evaluated between time points

^b^ according to authors impact trial, for all evaluated trials

^c^ impact was evaluated 61 times on 38 unique surgical trials

Medical records or hospital data and administrative databases were most often used as sources of data[27–34, 36–42, 44–57, 59, 62, 63]. Furthermore, most impact papers compared data before and after publication by performing a pre-trial and post-trial comparison, a trend analysis, or a mixture of those two methods. Five articles performed an interrupted time series analysis or spline regression analysis[31, 50, 52, 53, 57]. Five papers (14%) applied an economic evaluation by comparing total charges between time periods before and after trial publication[28, 37, 47, 50, 52]. Impact categories that were studied are outlined in Table 2.

**Table 2:** Analysis of findings from the surgical impact papers.

Impact category	No. of impact papers reporting on each type of impact	Impact found, yes (no, %)
Overall	61	49 (80)
Practice	19	15 (80)
Practice and policy	31	27 (87)
Practice and health gain	9	6 (67)
Practice, policy and health gain	2	1 (50)

All studies investigated changes in clinical practice, whereas some studies investigated changes in policy and health gain. RoB assessment of the impact papers is presented in Table 3. We appraised 1 study as ‘low RoB’[53], 5 studies as ‘moderate RoB’[37, 41, 47, 52, 56], 13 studies as ‘serious RoB’[28, 30–33, 39, 40, 43, 44, 48, 50, 57, 59], and 18 studies as ‘critical RoB’[27, 34–36, 38, 45, 46, 49, 51, 54, 55, 58, 60–65].

**Table 3:** Risk of bias assessment of impact papers (Robins-1).

Paper	C	SP	CI	DII	MD	OM	SR	Across domains
Adeoye	S	L	L	L	NI	M	S	S
Ahern	S	L	L	L	NI	M	S	S
Amin	S	L	L	L	NI	S	S	S
Baas	S	L	L	NI	S	C	M	C
Bazan	S	L	L	L	M	M	L	S
Beez	C	L	L	L	NI	S	C	C
Brown	L	L	L	L	L	S	L	S
Caudle	L	L	L	L	L	C	L	C
Colgan	C	M	L	L	NI	M	S	C
Costa	C	M	L	L	NI	S	C	C
Cox	C	L	L	L	NI	S	C	C
Degnan	C	L	L	L	M	S	C	C
Fillion	L	L	L	L	L	S	L	S
Gainer	C	L	L	NI	S	C	C	C
Garcia	L	L	L	L	L	M	L	M
Halm	S	L	L	L	M	S	L	C
Howard	M	L	L	L	NI	L	L	M
Hussain	L	L	L	L	L	L	L	L
Joyce	L	L	L	L	NI	S	C	C
Kelly	M	L	L	L	NI	M	L	M
Kirkman	M	L	L	L	NI	M	S	S
Knook	C	L	L	L	S	C	C	C
Le	L	L	L	L	L	C	L	C
Mahan	C	L	L	L	NI	C	C	C
Palmer	M	L	L	L	L	S	M	S
Potts	C	L	L	L	NI	S	C	C
Rea	L	L	L	L	L	M	L	M
Robinson	M	L	L	L	NI	M	C	C
Rosenbaum	M	L	L	L	NI	M	S	S
Rovers (‘03)	S	L	L	L	L	C	C	C
Rovers (‘09)	S	L	L	L	M	C	C	C
Salata	S	L	L	L	NI	L	S	S
Sheth	S	L	L	L	NI	L	L	S
Simon	C	M	L	L	NI	S	C	C
Smieliauskas	S	L	L	L	M	L	L	S
Williams	M	L	L	L	NI	M	L	M
Yao	S	L	L	L	NI	M	L	S

C: Confouding, SP: Selection of Participants, CI: Classification of Intervention, DII: Deviation of Intended Intervention, MD: Missing data, OM: Outcome Measurements, SR: Selective Reporting, L: Low Risk of Bias, M: Moderate Risk of Bias, S: Serious Risk of Bias, C: Critical Risk of Bias, NI: No Information on this domain.

### Conclusions of the impact papers

The impact of 7 surgical intervention trials was evaluated more than once[66–73], resulting in 61 conclusions by the authors concerning the impact of the trial paper, of which 49 times (80%) a significant impact on healthcare or policy was reported (S2 Table). In more recent years, a significant greater proportion of the articles reported impact on healthcare (*P* = 0.04) (Fig 2 and Table 5). Primarily, impact was found in a change in healthcare practice (mostly in a change in procedure rate after publication (n = 48, 98%)), but also in a change in policy e.g. a guideline revision (n = 17; 52%), and in a change in patient benefit, such as an increase or decrease in complications and mortality (n = 8; 24%). Additionally, 3 out of 5 papers that performed a cost evaluation reported cost savings[28, 50, 52] and 2 papers noticed a rise in healthcare costs[37, 47] after publication of the surgical trial.

The trial by Mendelow[70], that evaluated early surgery versus conservative treatment for intracerebral hemorrhage, was evaluated by 3 impact papers. Two of the impact papers reported a decrease in procedures[48, 49], whereas one paper did not observe a change in procedure rate[44]. However, this can be due to different study periods. The trial by Prinssen and by the EVAR trial participants[67, 74], that compared the effectiveness of endovascular aneurysm repair for abdominal aortic aneurysm with open repair, were evaluated twice[58, 59]. One paper did not find research impact by surveying Dutch surgeons before and after trial publication, while the other paper witnessed an increasing trend in numbers of endovascular procedures in the United Kingdom. For the remaining six papers that were examined more than once, no differences in conclusions were found between papers reporting on the same trial.

### Characteristics and quality assessment of the trial papers

Most trial papers were non-blinded multicenter RCTs (Table 4, details in S3 Table).

**Table 4:** Summary of the characteristics of the surgical intervention trial papers.

Characteristics	Evaluated trials (no. = 38)
Specialty, no. (%)	
*General surgery*	2 (5)
*Surgical oncology*	7 (18)
*Trauma surgery or orthopedic surgery*	7 (18)
*Vascular surgery*	12 (32)
*Otorhinolaryngology*	2 (5)
*Neurosurgery*	8 (21)
Publication period, no. (%)	
*1980–1989*	3 (8)
*1990–1999*	3 (8)
*2000–2009*	22 (58)
*2010–2014*	10 (26)
Study design, no. (%)	
*Cohort study*	1 (3)
*RCT*	36 (95)
*Meta-analysis*^*a*^	1 (3)
Multicenter trial, no (%), yes	30 (79)
International trial, no (%), yes	11 (29)
Blind RCT^b^, no (%), yes	5 (13)
Evaluation, no. (%)	
*Surgery vs*. *watchful waiting*	15 (39)
*Surgery vs*. *non-surgical treatment*	4 (11)
*Surgery vs*. *surgery*	19 (50)
Number of patients, median (IQR)	461 (131–991)
Economic evaluation, no (%), yes	16 (42)
Methods economic evaluation, no. (%)	
*Cost-effectiveness analysis (CEA)*	4 (11)
*Cost minimization analysis (CMA)*	2 (5)
*Cost utility analysis (CUA)*	10 (26)
External funding, no (%)	
*No external funding*	3 (8)
*Received external funding*	32 (84)
*Not mentioned*	3 (8)
Outcome according to author, no. (%)	
*No differences*	14 (37)
*Surgery is (cost)effective*	6 (16)
*Conservative is (cost)effective*	3 (8)
*One technique better than other*	10 (26)
*Different outcomes for different subgroups*	4 (11)
Implementation, no (%), yes	19 (50)
Risk of Bias, mean (SD)	21 (2.8)

^a^ Meta-analysis of randomized clinical trials with blinded outcome adjudication

^b^ Number of blind RCTs out of 33 RCTs

^c^ 16 surgical trials performed an (additional) economic evaluation

The median sample size was 461 (interquartile range (IQR): 131–991). Fifteen studies (39%) evaluated surgery vs. watchful waiting, while 19 studies (50%) evaluated surgery vs. surgery, and 4 studies (11%) evaluated surgery vs. a non-surgical treatment. The outcomes from the trials according to the authors were heterogeneous, and half of the papers supported de-implementation while the other half supported implementation of the evaluated procedure. The average score on the MINORS scale was 21 (SD 2.8).

### Determinants of impact

Outcomes on determinants of impact are shown in Table 5.

**Table 5:** Analysis of impact determinants.

Characteristics	Impact^a^	No impact^a^^,^^b^	p-value
	no. = 49 (%)	no. = 12 (%)	
Type of comparison trial paper			
*Surgery vs*. *surgery*	24 (49)	6 (50)	1.0
*Surgery vs*. *watchful waiting*	23 (47)	6 (50)	
*Surgery vs*. *non-surgical treatment*	2 (4)	0 (0)	
Significant difference found for treatment outcomes			
*Yes*	12 (25)	11 (92)	<0.001*
*No*	37 (76)	1 (8)	
Outcomes trial paper suggest:			
*Implementation*	17 (35)	9 (75)	0.02*
*De-implementation*	32 (65)	3 (25)	
Economic evaluation trial paper			
*Yes*	17 (35)	10 (83)	0.003*
*No*	32 (65)	2 (17)	
Surgical specialty			
*Surgical oncology*	14 (29)	0 (0)	0.05
*Other*	35 (71)	12 (100)	
External funding trial paper			
*No external funding*	4 (8)	1 (8)	1.0
*Received external funding*	42 (86)	11 (92)	
*Not reported*	3 (6)	0 (0)	
Sample size trial paper, median (IQR)	636 (131–991)	300 (145–995)	0.8
Time since trial paper publication^c^, median (IQR), m	127 (112–183)	176 (119–186)	0.2
Risk of Bias trial paper (MINORS)	21 (2.6)	22 (2.0)	0.3
Design impact paper			
*Descriptive*	13 (27)	5 (42)	0.4
*Comparative analysis*	25 (51)	6 (50)	
*Time series analysis*	11 (22)	1 (8)	
Data collection impact paper			
*Administrative database*	39 (80)	4 (33)	<0.001*
*Medical records*	9 (18)	3 (25)	
*Opinion of physicians*	1 (2)	5 (42)	
Case-mix presented in impact paper			
*Yes*	18 (37)	8 (67)	0.1
*No*	31 (63)	4 (33)	
Continent of impact evaluation			
*North America*	40 (82)	5 (42)	0.009*
*Europe*	9 (18)	7 (58)	
Timeframe impact evaluation (years), mean (SD)	6.1 (5.5)	11.0 (5.5)	0.02*
Time since publication impact paper^d^, months, median (IQR)	50 (33–88)	121 (39–158)	0.04*
Time between trial and impact paper, months, median (IQR)	91 (52–119)	57 (29–71)	0.05*
Risk of bias impact paper (Robins-1)			
*Low*	5 (10)	0 (0)	0.8
*Moderate*	5 (10)	1 (8)	
*Serious*	17 (35)	4 (33)	
*Critical*	22 (45)	7 (58)	

^a^ according to conclusion of authors

^b^ no impact or: no definite conclusions made by authors

^c^ months between literature search and publication date trial paper

^d^ months between literature search and publication impact paper

* statistically significant

Impact was found more often when impact was studies through administrative databases or medical records compared to through the opinion of physicians. Post-hoc analysis between the three groups showed a significant difference between the use of administrative data and the opinion of physicians (administrative database vs. opinion of physicians, *P*<0.001; medical records vs. opinion of physicians, *P =* 0.04). Additionally, impact papers from the continent of North America were more likely to report an impact on practice patterns than those from Europe. Correspondingly to Fig 2, more impact was found in more recent years (fewer months between our literature search and publication date of the impact paper). Also, a longer timeframe (in years) for impact evaluation was associated with finding impact. Additionally, more time (in months) between publication of the trial paper and publication of the impact paper lead to more healthcare impact. When no economic evaluation was performed additional to the trial paper, it was more likely that impact on healthcare was found. Furthermore, when the trial paper did not find a significant difference, the impact paper was more likely to find an impact. Additionally, we noticed that all surgical oncology papers (n = 14) translated research into practice, but this was not significantly different from other specialties. No differences were found for the other characteristics of the trial papers.

## Discussion

This systematic review of surgical impact papers found an increase in these published manuscripts over the years. Neurosurgical research and surgical oncology research was most often evaluated. However, of the large numbers of surgical trials that were published[14], only in a very small percentage the healthcare impact has been evaluated. Moreover, impact papers did not use frameworks, and results from the Risk of Bias assessment showed that many impact papers have a high RoB, which hampers the reduction of low-value surgical interventions and provision of ongoing feedback to decisionmakers[13, 75]. The analyses of impact determinants showed that certain methodological aspects of both the surgical trial papers and impact papers are advantageous for impact evaluation, such as a long enough timeframe to measure impact and the use of administrative databases compared with surveys assessing physician opinion.

### Impact frameworks

It is remarkable that not one of the identified impact papers mentioned the use of a framework to assess healthcare impact. In contrast, a review on multi-project research programs, including non-surgical projects, found that most impact papers did use a conceptual framework[23]. One explanation for this contrast could be that existing frameworks are designed for general research programs[8, 76–78], while, as described by the IDEAL recommendations, important differences exist between surgical intervention research and other research fields[17]. A general and specific approach for impact assessments in surgery, as an addition to the IDEAL framework, could improve methods and inform clinicians, researchers, and funding bodies.

### Importance of proper study design and data collection to evaluate healthcare impact of surgical trials

Our results showed that administrative databases and medical hospital data were most frequently used as data sources for surgical intervention research impact, and were more often associated with healthcare impact. In the IDEAL framework it is also recommended to use registries and routine databases for long-term study[79]. Not only is the use of administrative databases more objective than the opinion of experts, it might also be more representative for surgical research impact, as it includes a wider population and relatively longer follow-up is compared to hospital data[79, 80]. Conversely, data on specific case-mix variables is sometimes lacking within registers, which is important for proper comparison over time and between regions an which could be retrieved more easily in studies using patient charts.

We found more impact in more recent published impact papers, which might indicate more attention for research implementation in more recent years, but this could also indicate that more recent impact papers evaluated a longer time lag. Especially since the results show that impact could not have occurred yet within a limited time lag, and when not enough time has passed since publication of the trial paper: the implementation of 14% of all research into clinical practices takes 17 years on average[81–83]. Still, 80% of the trial papers in this review had an impact on clinical practice within an average time span of approximately 4 years. This might be explained by the fact that only pivotal, high quality surgical trials are selected for evaluation. The results showed that surgical impact papers were only published by authors from Europe or North America. Nevertheless, the largest increase in publication of randomized surgical intervention trials was observed in Asia, implying more surgical impact assessments are needed there[15]. Additionally, the results showed that impact papers from North America found more often an impact on healthcare than those from Europe. This could indicate that practice in North America is more susceptible to research, but it could also be that researchers in Europe are more likely to study and publish about studies with an unclear research impact. We found only one paper that focused on impact in terms of changes in geographic variation. Since it was suggested that practice variation can partly be explained by gaps in scientific knowledge, future research could also focus on evaluating impact on practice variation[84].

### Analysis of impact

In this review impact on healthcare practice was found in most of the papers. However, it is important to assess the impact of published trials independent of already existing time-trends in the frequency of treatment, in treatment approach, or both[85]. Unfortunately, this was only performed in the minority of studies. One possibility to correct for time-trends is the use of difference in difference analysis[86]. The ideal control group would be a group that is unaware of a certain trial publication, but randomizing for the knowledge of trial results would be impossible and unethical. One option could be to compare with another intervention which was not evaluated. Another possibility could be performing interrupted time series analysis and to control for secular trends in the data by using segmented regression to measure the changes in procedures before and after trial publication[86, 87]. Three impact papers showed data that were measured after publication only, although impact studies require a comparative study[85, 88]. Hopefully, it now is easier to perform comparative studies with the rise and availability of multiple healthcare administrative databases. In addition, we found limited numbers of studies that analyzed costs before and after trial publication. The authors feel cost analyses, for example return-on-investment analysis or cost-benefit analysis, could be beneficial in the impact assessment of surgical research, especially since huge savings from reducing low-value surgery were predicted[13].

### Trial paper determinants of impact

More frequently impact was reported in cases of trials that did not find statistically significant differences, although from previous research the opposite was concluded[89, 90]. However, especially in the surgical field there is special attention for reducing low-value interventions[13] which might support this result. When no differences are found between an interventional procedure and watchful waiting for instance, one can say that this intervention is of low value and a change in procedure numbers is expected[13]. Indeed, a majority of the surgical trial papers supported de-implementation of a surgical technique, which was also a determinant for finding research impact. Furthermore, more impact papers found impact when no additional economic analysis was performed on the original trial; although in most cases the economic evaluation supported the outcomes from the RCT, making the evidence even stronger. It might be difficult to publish an additional economic analysis, when strong evidence on the effectiveness of a surgical intervention is already published and widely accepted, which we observe in impact on healthcare. Although not significant, it is notable that all surgical oncology impact papers reported impact on healthcare. This implies more attention for research or evidence-based medicine in the surgical oncology field compared to other surgical specialties.

### Ideas on improvement of knowledge translation in the current era

In this review, we focused on the impact of surgical research, which can support prompter implementation and thereby improve quality of healthcare. In the Dutch program ‘Leading the change’, five factors that influence implementation were identified, of which one is the use of audit and feedback for healthcare quality evaluation[91]. Encouragement on the use of impact evaluations by governments and funding bodies is needed to address the importance of these studies. More research on methodological issues and reporting guidelines for healthcare evaluations is needed to provide universal guidelines for research impact evaluations. Also, more research is needed on why some study results are translated into clinical practice whereas other results are not. It would also be interesting to investigate the impact of research on regional variation in healthcare as stated in the IDEAL-framework[92]. Moreover, it is believed that little variation is seen in clinical practice when there is strong evidence and a professional consensus for interventions[93].

### Strengths and limitations

To our knowledge, the present review is the first that specifically focuses on the impact of surgical research. This is necessary since there are some inherent differences with non-surgical studies and therefore different approaches to evaluate research impact are needed for both research fields[5, 19, 21]. Additionally, a limitation of this review is the small numbers of papers reporting ‘no impact’, which impeded multivariate analyses. Despite the focus on surgical trials, we found heterogeneous outcomes and evaluated procedures, which may have hidden the influence determinants can have in a more homogeneous setting. Last, previous reviews on methodological frameworks for research impact mentioned that they found parts of their included publications through grey literature (papers not indexed in bibliographic databases)[7, 20, 21]. This might be similar for impact papers since it is a relatively new research field, resulting in an underestimation of the number of surgical trial papers.

## Conclusions

In conclusion, more impact papers are needed to track changes in healthcare practice over time and provide knowledge on the impact of surgical research to researchers, funders, physicians, and policy makers. Eventually, this knowledge can help to reduce low-value surgical procedures. However, quality improvement of the used methods of published impact papers is necessary to draw valid conclusions, especially since we found that timeframe of evaluation and the data source of the impact papers is associated with finding research impact. We advise to collect data from either medical records or administrative databases, and perform comparative studies with a time frame of at least 4 years after publication. By routinely using valid methods as a completion of stage 4 of the IDEAL-framework, knowledge on societal research impact can be demonstrated and thereby feedback on overall quality of care.

## Supporting information

S1 Checklist PRISMA 2009 checklist. (DOC)

S1 Table Extensive information on impact papers. (DOCX)

S2 Table Extensive information on trial papers. (DOCX)

S3 Table Risk of Bias surgical intervention trial papers. (DOCX)

S1 File Literature search. (DOCX)
