# Supplementary material for: Impact of surgical intervention trials on healthcare: A systematic review of assessment methods, healthcare outcomes, and determinants
Source: PLoS One. 2020 May 22;15(5):e0233318. doi: 10.1371/journal.pone.0233318 (PMC7244162; doi:10.1371/journal.pone.0233318)
Supplement: S1 Table — (DOCX) [file pone.0233318.s002.docx]

**Table 6.** **Extensive information on research impact papers**

| Author  (year) | Specialty | Region | Data collection methods | Time frame (years) | Impact category | Outcome measurement | Analysis methods | Limitations mentioned in paper |
| --- | --- | --- | --- | --- | --- | --- | --- | --- |
| Adeoye (2010) | neurosurgery | USA | retrospective review of discharges from the Premier Hospital Database | initial trial: 2005  data: 2000-2008 | policy, practice, health gain | no. of procedures, mortality | - before/after trial  - baseline  - trend analysis corrected for age, race, and gender | reliance on an administrative database, which leads to the missing of detailed clinical data |
| Ahern  (2008) | surgical oncology | Northern Denmark | retrospective review of hospital discharge registries of three Danish counties and Danish cancer registry | initial trials: 1981, 1985, 1988  data: 1982-2002 | policy, practice | no. of procedures, age, and disease stage | - Veronesi:  after trial  - Fisher/Blichert: before and after trial  - baseline  - trend presented for age and disease stage groups: eye-balling | not mentioned |
| Amin  (2017) | orthopedic surgery | USA | retrospective review of State Ambulatory Surgery Database | initial trial: 2002  data: 1998, 2000, 2002, 2004, and 2006 | practice | no. of procedures, diagnosis, age | - before/after trial  - baseline  - case-mix presented pre-post analysis  - trend: eye-balling | limited number of states, no surgeon-specific factors or trends among individual surgeons |
| Baas  (2007) | vascular surgery | the Netherlands | questionnaire: n=1,400 Dutch surgeons and trainees (16% response) | initial trials: 2004, 2005  data: 2005 | practice | pre- and post-trial beliefs concerning treatment choice, role and fields of interest of respondents | - after trial  - descriptive | it might be hard to remember pre-trial opinions, since the survey is executed 6 years after the trial. Low response rate |
| Bazan (2019) | surgical oncology | Ohio, USA | retrospective review of Surveillance Epidemiology and End Results registry data | initial trial: 2004  data: 2000-2014 | policy, practice | no. of procedures, patient and tumor characteristics | - before/after trial  - trend: eye-balling | the retrospective design limits data on the influence of patient choice and certain clinical and pathological variables |
| Beez  (2017) | neurosurgery | Düsseldorf, Germany | retrospective review of hospital data from one hospital | initial trials: 2007, 2009  data: 2000-2017 | policy, practice | no. of procedures | - before/after trial  - trend: eye-balling | no subgroup analyses |
| Brown (2009) | vascular surgery | Leicester, UK | prospectively maintained database from one hospital in Leicester | initial trial: 2005  data: 2000-2006 | practice, health gain | no. of procedures, mortality, complications, sex, age, comorbidity | - before/after trial  - case-mix presented pre-post analysis  - trend: eye-balling | not mentioned |
| Caudle (2012) | surgical oncology | Texas, USA | retrospective review of medical records at a cancer center | initial trial: 2010  data: 04-2009 to 08-2011 | policy, practice, | no. of procedures, patient age, menopausal status, tumor and lymph node characteristics | - before/after trial  - case-mix presented pre-post analysis | the retrospective nature limits the analysis concerning decision making |
| Colgan (2019) | trauma surgery | Ireland | retrospective review of Irish Hospital In-Patient Enquiry data | initial trial: 2014  data: 2008-2017 | practice | no. of procedures, training vs. non-training hospitals | - before/after trial  - trend: eye-balling | no specific diagnosis codes for all techniques in database, other trials influence decision making |
| Costa  (2016) | trauma surgery | UK | retrospective review of the NHS English Hospital data | initial trial: 2014  data: 2005-2015 | practice | no. of procedures, hospital | - before/after trial  - trend: eye-balling | no specific diagnosis code in administrative database, evaluation of one country only. |
| Cox  (2016) | neurosurgery | USA | retrospective review of Medicare Database | initial trials: 2009, 2010  data: 2006-2013 | policy, practice | no. of procedures, specialty group | - before/after trial  - descriptive  - trend: eye-balling | not mentioned |
| Degnan (2017) | neurosurgery | USA | retrospective review on Medicare claims | initial trials: 2009  data: 2001-2014 | policy, practice | no. of procedures, specialty | - before/after trial  - descriptive  - trend: eye-balling | no long-term benefits measured, no causality can be inferred from an observational study |
| Fillion  (2017) | surgical oncology | Columbus, USA | retrospective review of hospital data from one institution | initial trial: 2010  data: 2007-2013 | policy, practice, | no. of procedures, patient and tumor characteristics, costs | - before/after trial  - case-mix presented pre-post analysis  - trend: eye-balling  - cost evaluation | retrospective design, anesthesia and outpatient costs not included |
| Gainer (2012) | surgical oncology | USA | questionnaire: n=2759 Breast Surgeons (30.8% response) | initial trial: 2010  data: 07/08-2011 | policy, practice | surgeon’s characteristics, familiarity with results, and treatment choice | - after trial  - descriptive | brief survey, limited to seven questions with simple scenarios without specification of patient characteristics |
| Garcia-Etienne (2019) | surgical oncology | Italy, Germany, Belgium, Switzerland, Austria, the Netherlands | retrospective review of the European Society of Breast Cancer Specialists data | initial trial: 2010  data: 2005-2016 | policy, practice | no. of procedures, geographic differences | - before/after trial  - trend analysis corrected for age, country, and tumor characteristics | use of administrative data |
| Halm  (2007) | vascular surgery | New York state, USA | retrospective review of Medicare claims (US) and the NY state hospital discharge database | initial trials: 1998  data: 01-1998 to 06-1999 | policy, practice | no. of procedures, disease stage, comorbidity | - before/after trial  - baseline  - pre-post analysis on no. of procedures over time and ratings of appropriateness compared with a pre-trial study[60] | expert opinion for appropriateness ratings is subjective and susceptible to bias |
| Howard (2012) | orthopedic surgery | Florida, USA | retrospective review of Florida's State Database | initial trials: 2002, 2008  data: 1998-2010 | policy, practice | no. of procedures, costs | - before/after trial  - spline regression analysis corrected for age, sex  - cost evaluation | not mentioned |
| Hussain (2016) | vascular surgery | Ontario, Canada | retrospective review of Linked population-level Healthcare databases | initial trials: 2004, 2006, 2010  data: 2002- 2014 | practice | no. of procedures, diagnosis, and specialty | - before/after trial  - baseline  - interrupted time series analysis controlled for age, sex | small study time, prior procedures are not specified, limited to one area in Canada |
| Joyce  (2015) | surgical oncology | Cork, Waterford, Dublin, Ireland | prospectively maintained national database from three tertiary referral centers | initial trial: 2010  data: 2010-2012 | policy, practice | no. of procedures, tumor characteristics, patient age | - before/after trial  - case-mix presented pre-post analysis | not mentioned |
| Kelly  (2014) | neurosurgery | USA | retrospective review of the Nationwide Inpatient Sample from non-federal US hospitals | initial trial: 2005  data: 2000-2010 | practice, health gains | no. of procedures, race, SES, comorbidities, hospital, diagnosis, outcome, and costs | - before/after trial  - case-mix presented pre-post analysis  - trend: eyeballing | long-term trends were not measured, clinical information may be important for decision-making |
| Kirkman (2008) | neurosurgery | Newcastle, UK | retrospective review of electronic records from four hospitals in Newcastle | initial trial: 2005  data: 2002, 2004, 2006, and 2007 | practice, health gains | no. of procedures, no. of admissions, mortality, sex, age | - before/after trial  - baseline  - trend analysis | outcomes are limited to the hospitals of Newcastle, diagnosis coding is conducted by non-clinical office workers |
| Knook (2001) | general surgery | the Netherlands | questionnaire: n=780 Dutch surgeons (100% response) | initial trial: 1997  data: 1998 | practice | no. of procedures, preferred technique, diagnosis, type of hospital, reasons for not performing the technique | - after trial  - descriptive | missing separate registration codes for endoscopic procedures |
| Le  (2016) | surgical oncology | Greenville, USA | prospectively maintained database from one hospital | initial trial: 2010  data: 2009-2013 | practice | no. of procedures, patient age, tumor characteristics | - before/after trial  - baseline  - case-mix presented pre-post analysis | long-term effects are not measured |
| Mahan (2012) | orthopedic surgery | Boston, USA | retrospective review of operative and clinical notes from 8 surgeons | initial trial: 2007  data: 2000-2009 | practice | no. of procedures, complications | - before/after trial  - case-mix presented pre-post analysis | retrospective data collection, same surgeons pre-trial and post-trial; results can be due to more experience |
| Palmer (2017) | surgical oncology | Charlotte, USA | retrospective review of hospital data from one hospital | initial trial: 2010  data: 2008-2014 | practice, health gains | no. of procedures, patient age, tumor characteristics | - before/after trial  - case-mix presented pre-post analysis  - trend: eye-balling | short follow-up time, data might be influenced by other practice patterns indications or administration |
| Potts  (2012) | orthopedic surgery | USA | retrospective review of The American Board of Orthopedic Surgery database | initial trial: 2002  data: 1999-2009 | policy, practice | no. of procedures, diagnosis | - before/after trial  - trend analysis  - checked for differences in diagnosis, age and sex | the group of surgeons is not necessarily representative, coding is surgeon dependent, impossible to evaluate diagnosis |
| Rea  (2011) | surgical oncology | USA | retrospective review of the Healthcare Cost and Utilization Project's nationwide inpatient sample | initial trial: 2004  data: 2001-2007 | policy, practice, health gains | no. of procedures, diagnosis, sex, age, place of residence, comorbidity, insurance, hospital, and costs | - before/after trial  - case-mix presented pre-post analysis  - trend analysis | miscoding may occur due to the use of an administrative database, no operative details can be obtained and no data after discharge, no outpatient data. |
| Robinson (2014) | surgical oncology | Phoenix, USA | prospectively collected database from one hospital | Initial trial: 2010  data: 2003-2013 | policy, practice | no. of procedures, age, tumor characteristics | - before/after trial  - baseline  - pre-post analysis | not mentioned |
| Rosenbaum (2017) | neurosurgery | USA | retrospective review of Nationwide Inpatient Sample | initial trials: 2009  data: 2005-2011 | practice, health gains | no. of procedures, diagnosis, patient age, sex, race, comorbidity, insurance, hospital, and admission characteristics | - before/after trial  - case-mix presented pre-post analysis  - trend: eye-balling | reliance on administrative coded data, no outpatient data |
| Rovers (2003) | ENT-surgery | the Netherlands | questionnaire: n=75 ENT-surgeons (69.3% response) | initial trial: 2001  data: unclear | practice | pre- and post-trial beliefs concerning treatment choice | - before/after trial  - pre-post analysis on treatment choice | not possible to measure individual changes in beliefs, non-response rate, information might be insufficient |
| Rovers (2009) | ENT-surgery | the Netherlands | questionnaire: n=120 ENT-surgeons (63% response for pre-trial beliefs, 41% posterior), n=120 GPs (58% response for pre-trial belief, 38% posterior) | initial trial: 2004  data: 2002, 2006 | policy, practice | pre- and post-trial beliefs concerning treatment choice | - before/after trial  - baseline  - pre-post analysis on treatment choice | information may be insufficient, high nonresponse rate, differences pre- and post-trial beliefs not compared |
| Salata (2019) | vascular surgery | Ontario, Canada | retrospective review of data from the Institute for Clinical and Evaluative Sciences | initial trials: 2010, 2012  data: 2002-2016 | practice | no. of procedures | - before/after trial  - interrupted time series analysis | indirect identification of patients using codes |
| Sheth  (2017) | orthopedic surgery | Ontario, Canada | retrospective review of linked population-level healthcare databases Ontario | initial trial: 2010  data: 2002-2014 | practice | no. of procedures | - before/after trial  - baseline  - interrupted time series analysis | codes for ruptures of Achilles tendon are not validated, Ontario is the region of primary investigators, changes in fees are not included |
| Simon (2015) | neurosurgery | USA | retrospective review of the administrative Medicare database in the USA | initial trials: 2002, 2005, 2009  data: 1996-2013 | Practice | no. of procedures | - before/after trial  - trend analysis | bias through changing proportions of specialists, reliance on administrative dataset |
| Smieliauskas (2014) | neurosurgery | Florida, USA | retrospective review of Florida inpatient hospital data and population counts | initial trials: 2009  data: 2005-2012 | policy, practice | no. of procedures, specialty, costs | - before/after trial  - baseline  - interrupted time series analysis corrected for patient age, sex, site of care, payer, and diagnosis  - cost evaluation | analysis restricted to Florida, other possible confounders not included (e.g. recession in 2007), and procedures performed in physician office settings not included |
| Williams (2014) | general surgery | Memphis and Nashville, USA | retrospective review of data collected from the trial hospital | initial trial: 2011  data: 2005-2011 | practice, health gains | no. of procedures, age, sex, race, insurance, diagnosis, complications | - before/after trial  - case-mix presented pre-post analysis | other differences in treatment, costs, and long term functional status were not considered, single institution analysis |
| Yao  (2015) | surgical oncology | USA | retrospective review of the National Cancer database | initial trial: 2010  data: 1998-2011 | policy, practice | no. of procedures, patient age, race, tumor characteristics, hospital | - before and after  - baseline  - case-mix presented pre-post analysis  - trend analysis | study consists of a select cohort of patients |
