# Supplementary material for: Impact of surgical intervention trials on healthcare: A systematic review of assessment methods, healthcare outcomes, and determinants
Source: PLoS One. 2020 May 22;15(5):e0233318. doi: 10.1371/journal.pone.0233318 (PMC7244162; doi:10.1371/journal.pone.0233318)
Supplement: S2 Table — (DOCX) [file pone.0233318.s003.docx]

**Table 7.** **Extensive information on investigated trials**

| Author  (year) | Comparison | Sample size | Economic evaluation | R/M/O^b^ | Funding | Conclusion^a^ | Impact paper (year), region | Main results | Impact^a^ |
| --- | --- | --- | --- | --- | --- | --- | --- | --- | --- |
| Blakely  (2011) | early vs. interval appendectomy | 131 | CMA | R | no external funding | Early appendectomy significantly reduced adverse event rates, costs, and time away from normal activities. | Williams (2014), Memphis and Nashville, USA | 67% of the pretrial patients underwent early surgery, 84% of post-trial patients did (*p*=.005). Pretrial patients had more adverse events (*p*=.02). | unclear |
| Blichert-Toft (1988) | breast-conserving surgery  (BCS) vs. radical mastectomy | 847 | n.a. | R | received external funding | no differences | Ahern (2008), Northern Denmark | Between 1982 and 2002 there is an upward trend in the proportion of BCS from 1% to 25%. | yes |
| Boughey (2013) | Sentinel lymph node dissection (SLND) vs. axillary lymph node dissection  (ALND) | 756 | n.a. | O | received external funding | The use of SLND is supported as an alternative to ALND in a subset of women. | Palmer (2017), Charlotte, USA | 73% of post-trial cohort had SLNB surgery, whereas 1% of the pre-trial cohort had. Pre-trial 99% had ALND surgery, post-trial only 27% did. | yes |
| Brott  (2010) | carotid endarterectomy  (CEA) vs. carotic artery stenting (CAS) | 2502 | CUA | R^d^ | received external funding | no differences | Hussain (2016), Ontario, Canada | The rate of endarterectomy did decrease after publication in 2010 (*p*=.005). | yes |
| de Bruin (2010) | conventional open repair (OR) vs. EVAR | 351 | n.a. | R | received external funding | no differences | Salata (2019), Ontario, Canada | A decrease in the rate of open repair was found after publication of the trial in 2010 (p=0.01), the uptake of EVAR was unaffected (p=0.14). | yes |
| Buchbinder (2009) | vertebroplasty vs. conservative treatment | 78 | CUA | R^d^ | received external funding | no differences | Cox (2016), USA | Number of procedures decreased from 101,807 in 2008 to 80,940 in 2013, with a sharp decrease after 2009. | yes |
|  |  |  |  |  |  |  | Degnan (2017), USA | Numbers of vertebroplasty increased from 18,911 in 2001 to 38,123 in 2008, and decreased from 2008 to 2014 (14,898). | yes |
|  |  |  |  |  |  |  | Rosenbaum (2017), USA | Procedural rate decreased from 20% (2005-2008) to 15% (2010-2011) (*p*<.0001). | yes |
|  |  |  |  |  |  |  | Simon (2015), USA | Number of procedures decreased from 99,961 in 2009 to 77,108 in 2013. | yes |
|  |  |  |  |  |  |  | Smieliauskas (2014), Florida, USA | Vertebroplasty rate declined 52%, and kyphoplasty rate declined 40% (*p*<.01), implying savings of over $1 billion per year. | yes |
| Carotid Stenting Trialists’ Collaboration (2010) | CEA vs. CAS | 3433 | n.a. | M | received external funding | CEA gives lower rates of stroke and death compared to CAS, especially in older patients (age≥70 years). | Hussain (2016), Ontario, Canada | The rate of endarterectomy did decrease after publication in 2010 (*p*=.005). | yes |
| Costa  (2014) | kirschner wires vs. volar locking plate fixation | 461 | CUA | R | received external funding | No differences in outcomes, Kirschner wires are cheaper compared to volar locking plates. | Costa (2016), USA | 2005-2010: internal fixation 75% vs. K-wire 12% vs. other types 13%. 2014: internal fixation 42% vs. K-wire 48% vs. other 10%. | yes |
|  |  |  |  |  |  |  | Colgan (2019), Ireland | The rate of K-wire fixation declined from 59% in 2008 to 30% in 2017, while the rate of plate fixation increased from 56% to 62%, subsequently. | yes |
| European Carotid Surgery Trialists’ Collaborative (1998) | CEA vs. conservative treatment | 3024 | n.a. | R | received external funding | Surgery is indicated for patients with symptomatic stenosis > 70-80%. | Halm (2007), New York state, USA | 87% (after publication) vs. 35% (before) of operations were done for appropriate reasons, 4% vs. 32% for uncertain reasons, and 9% vs. 32% for inappropriate reasons (*p*<.0001). | yes |
| EVAR trial participants (2005) | OR vs. EVAR | 1082 | CUA | R | received external funding | No differences in mortality and quality of life, EVAR gives more complications and re-interventions and is more expensive than OR. | Baas (2007), the Netherlands | Before publication, 72% of the surgeons preferred OR for patients without comorbidity, after 74% did. Before the trial 76% preferred EVAR for patients with comorbidity, after 74% did. | no |
|  |  |  |  |  |  |  | Brown (2009), Leicester, UK | The proportion of EVARs was 52% one year before publication vs. 57% one year after (*p*=.64), however between 2000 and 2006 the overall percentage increased from 28% to 57%. | unclear |
| Fisher  (1985) | total mastectomy vs. segmental mastectomy | 2163 | n.a. | R | received external funding | no differences | Ahern (2008), Northern Denmark | Between 1982 and 2002 there is an upward trend in the proportion of BCS from 1% to 25%. | yes |
| Greenhalgh (2010) | OR vs. EVAR | 1252 | CEA | R | received external funding | lower operative mortality and lower costs with EVAR compared to OR, but no differences in total mortality | Salata (2019), Ontario, Canada | A decrease in the rate of open repair was found after publication of the trial in 2010 (p=0.01), the uptake of EVAR was unaffected (p=0.14). | yes |
| Giuliano  (2010) | ALND and SLND vs. SLND | 991 | n.a. | R | received external funding | no differences | Caudle (2012), Texas, USA | Post-trial indication for surgery were adjusted to conclusions of the Z0011 trial. Pre-trial 85% underwent surgery, post-trial 24% did (*p*<.001). | yes |
|  |  |  |  |  |  |  | Fillion (2017), Columbus, USA | No significant differences in patient and tumor characteristics. Reduction in surgery from 78% to 21% (*p*<.001). Costs savings were estimated at $203.187. | yes |
|  |  |  |  |  |  |  | Gainer (2012), USA | The majority (97%) of the respondents were familiar with the trial. 57% would almost never perform surgery. | yes |
|  |  |  |  |  |  |  | Garcia-Etienne (2019), Italy, Germany, Belgium, Switzerland, Austria, the Netherlands | A decrease in number of ALNDs is observed (89% in 2010 to 73% in 2016), but with differences between countries. | yes |
|  |  |  |  |  |  |  | Joyce (2015), Cork, Waterford, Dublin, Ireland | A significantly lower number of ALNDs were performed in the post-trial cohort (71% versus 94% in the pre-trial cohort, *p*=.0022), of the patients who fitted to the study eligibility, in the pre-trial cohort 92% underwent ALND compared to 66% in the post-trial cohort (*p*=.0006). | yes |
|  |  |  |  |  |  |  | Le (2016), Greenville, USA | 79% of pre-trial patients underwent additional surgery (lymph node dissection), compared with 52% in the post-trial group (*p*<.001). | yes |
|  |  |  |  |  |  |  | Robinson (2014), Phoenix, USA | Before trial presentation 84% of all patients underwent a procedure; afterward only 63% did (*p*<.01). Similar results were found before and after publication. | yes |
|  |  |  |  |  |  |  | Yao (2015), USA | An increase is seen in procedure rates between 2010 and 2011 (20.2 - 31% for the different types of diagnoses). | yes |
| Hofmeijer (2009) | Surgical decompression vs. conservative treatment | 64 | n.a. | R | received external funding | Surgery reduces poor outcomes compared to conservative treatment. | Beez (2018), Düsseldorf, Germany | Between 2009 and 2015 number of procedures remained stable with median of 45 operations per year. | no |
| Hughes (2004) | Lumpectomy and breast radiotherapy vs. mastectomy | 636 | n.a. | R | received external funding | Lumpectomy yields equivalent cancer outcomes to mastectomy in women ≥ 70 years | Bazan (2019), Ohio, USA | Mastectomy rates decreased from 29% before 2004 to 23% after 2004, mostly in non-hispanic white women | yes |
| Juttler  (2007) | Surgical decompression vs. conservative treatment | 32 | n.a. | R | no external funding | Surgical decompression reduces mortality in large strokes compared to conservative treatment. | Beez (2018), Düsseldorf, Germany | Between 2008 and 2009 numbers of procedures increased from 26 to 43 per year and remained stable afterwards. | yes |
| Kallmes  (2009) | vertebroplasty vs. conservative treatment | 131 | CUA | R^d^ | received external funding | no differences | Cox (2016), USA | Number of procedures decreased from 101,807 in 2008 to 80,940 in 2013, with a sharp decrease after 2009. | yes |
|  |  |  |  |  |  |  | Degnan (2017), USA | Numbers of vertebroplasty increased from 18,911 in 2001 to 38,123 in 2008, and decreased from 2008 to 2014 (14,898). | yes |
|  |  |  |  |  |  |  | Rosenbaum (2017), USA | Procedural rate decreased from 20% (2005-2008) to 15% (2010-2011) (*p*<.0001). | yes |
|  |  |  |  |  |  |  | Simon (2015), USA | Number of procedures decreased from 99,961 in 2009 to 77,108 in 2013. | yes |
|  |  |  |  |  |  |  | Smieliauskas (2014), Florida, USA | Vertebroplasty rate declined 52%, and kyphoplasty rate declined 40% (*p*<.01), implying savings of over $1 billion per year. | yes |
| Kirkley  (2008) | arthroscopy vs. conservative treatment | 188 | n.a. | R^d^ | not reported | no differences | Howard (2012), Florida, USA | Number of arthroscopies decreased 47 percent between 2001 and 2010 (*p*<.001). | yes |
| Klazen  (2010) | vertebroplasty vs. conservative treatment | 202 | CUA | R | received external funding | Vertebroplasty is superior to conservative treatment for pain relief. | Cox (2016), USA | Number of procedures decreased from 101,807 in 2008 to 80,940 in 2013. | no |
| Kocher  (2007) | lateral entry vs. medial and lateral entry | 217 | n.a. | R | received external funding | no differences | Mahan (2012), Boston, USA | Pre-trial medial and lateral vs. lateral entry was 83 vs. 58, post-trial this was 16 vs. 110 (*p*<.001). | yes |
| Lederle (2012) | OR vs. EVAR | 881 | CEA | R | received external funding | no differences in mortality in older patients, but lower costs with EVAR | Salata (2019), Ontario, Canada | The trial did not result in a change in the rate of one of both procedures (OR: p = 0.09, EVAR: p = 0.56). | no |
| Liem  (1997) | laparoscopic vs. open inguinal repair | 110 | CEA | R | received external funding | Laparoscopic showed better results than conventional repair and is found less expensive. | Knook (2001), the Netherlands | Laparoscopic hernia repair was performed by 16% of Dutch surgeons in 1998, open repair by 84%. | no |
| Mas  (2006) | CEA vs. CAS | 527 | n.a. | R | received external funding | Rates of death and stroke were lower with CEA compared to CAS. | Hussain (2016), Ontario, Canada | After 2006 the CEA rate decreased (*p*=.04). The rate of CAS did not change (*p*=.11 and *p=*.34). | yes |
| Mendelow (2005) | early surgery vs. watchful waiting in patients with intracerebral hemorrhage (ICH) | 1033 | n.a. | R | received external funding | no differences | Adeoye (2010), USA | No significant change in rate of surgery after publication (*p*=.015). | no |
|  |  |  |  |  |  |  | Kirkman (2008), Newcastle, UK | Surgical procedures decreased from 32% in 2002 to 17% in 2007 (χ^2^=11.919, *p*=.008, df=3). | yes |
|  |  |  |  |  |  |  | Simon (2015), USA | Total number of procedures decreased from 2341 in 2002 to 1646 in 2011 (*p*=.03). | yes |
| Molyneux (2002) | neurosurgical clipping vs. endovascular coiling | 2143 | n.a. | R | received external funding | Endovascular coiling gives a survival benefit for at least 7 years compared to neurosurgical clipping. | Simon (2015), USA | Before publication an increase in coiling of 4% is seen, after publication this was 6% until 2007 (*p*=.01). | yes |
| Moseley  (2002) | arthroscopy vs. placebo surgery for patients with osteoarthritis of the knee | 180 | n.a. | R^d^ | no external funding | no differences | Amin (2017), USA | The number of knee arthroscopies increased from 155,057 per year in 1998 to 172,317 in 2006 (*p*≤.001), mean age increased from 47.6 to 49.2 years (*p*<.001), and the indication osteoarthritis decreased from 11% to 7% (*p*<.001). | yes |
|  |  |  |  |  |  |  | Howard (2012), Florida, USA | Number of arthroscopies decreased 47 percent between 2001 and 2010 (*p*<.001), and a reduction of $82-$138 million per year. | yes |
|  |  |  |  |  |  |  | Potts (2012), USA | Knee arthroscopy decreased from a peak of 8418 cases in 2001 to 5923 cases in 2009. | yes |
| North American symptomatic carotid endarterectomy trial collaborators (1998) | CEA vs. conservative treatment | 2267 | n.a. | R | not reported | surgery is indicated for patients with symptomatic stenosis > 70%, patients with stenosis <50% did not benefit from surgery. | Halm (2007), New York state, USA | 87% (after publication) vs. 35% (before) of operations were done for appropriate reasons, 4% vs. 32% for uncertain reasons, and 9% vs. 32% for inappropriate reasons (*p*<.0001). | yes |
| Nelson  (2004) | laparoscopic vs. open colectomy | 872 | n.a. | R | received external funding | no differences | Rea (2011), USA | Percentages of laparoscopic colectomy has increased from 6% in 2001-2003 to 12% in 2005-2007 for benign disease, and from 2% to 9% for colon cancer. Charges increased $7700 per hospitalization for those time periods. | yes |
| Patchell  (2005) | decompressive surgery and radiotherapy vs. radiotherapy alone | 101 | n.a. | R | received external funding | decompressive surgery is superior to the non-surgical treatment. | Kelly (2014), USA | After publication, an increase in surgery from 4% to 5% is seen. A rise in costs is seen from $62.078 pre-RCT to $107.298 post-RCT. | yes |
| Prinssen  (2004) | conventional open repair (OR) vs. EVAR | 345 | CUA | R | received external funding | EVAR has lower mortality rates than OR for large aneurysms, however EVAR showed no QALY gain and an increase in costs. | Baas (2007), the Netherlands | Before publication, 72% of the surgeons preferred OR for patients without comorbidity, after 74% did. Before the trial 76% preferred EVAR for patients with comorbidity, after 74% did. | no |
| Rousing  (2009) | vertebroplasty vs. conservative treatment | 50 | CUA | R | received external funding | no differences | Degnan (2017), USA | Numbers of vertebroplasty increased from 18,911 in 2001 to 38,123 in 2008, and decreased from 2008 to 2014 (14,898). | yes |
|  |  |  |  |  |  |  | Rosenbaum (2017), USA | Procedural rate decreased from 20% (2005-2008) to 15% (2010-2011) (*p*<.0001). | yes |
| Rovers  (2001) | ventilation tubes vs. conservative treatment | 187 | CMA | R | received external funding | Treatment with ventilation tubes results in higher costs without clinical benefits compared to watchful waiting. | Rovers (2003), the Netherlands | Expectations of ventilation tubes remained high after the trial for the best-case as well as the worst-case scenario. | no |
| SPACE collaborative group  (2006) | CEA vs. angioplasty | 1183 | n.a. | R | received external funding | no differences | Hussain (2016), Ontario, Canada | After 2006 the CEA rate decreased (*p*=.04). The rate of CAS did not change (*p*=.11 and *p=*.34). | yes |
| Van Staaij (2004) | ATE vs. conservative treatment | 300 | CEA | R | received external funding | ATE gives an increase in costs without clinical benefits compared to no surgery. | Rovers (2009), the Netherlands | Prior and posterior beliefs did not differ and expectations of ATE remained high. | No |
| Vahedi  (2007) | surgical decompression vs. conservative treatment | 38 | n.a. | R | received external funding | surgical decompression reduces mortality compared to conservative treatment. | Beez (2018), Düsseldorf, Germany | Between 2008 and 2009 numbers of procedures increased from 26 to 43 per year and remained stable afterwards. | yes |
| Veronesi (1981) | BCS vs. radical mastectomy | 925 | n.a.^c^ | R | not reported | no differences | Ahern (2008), Northern Denmark | Between 1982 and 2002 there is an upward trend in the proportion of BCS from 1% to 25%. | yes |
| Wardlaw (2009) | kyphoplasty vs. conservative treatment | 300 | CUA | R | received external funding | Surgery is effective and safe, no conclusion about cost-effectiveness. | Rosenbaum (2017), USA | Procedural rate decreased from 20% (2005-2008) to 15% (2010-2011) (*p*<.0001). | No |
| Willits  (2010) | operative vs. conservative treatment of acute Achilles tendon ruptures | 144 | n.a. | R | received external funding | no differences | Sheth (2017), Ontario, Canada | By the end of the study period the observed rate was 6.5 operations per 100 cases (predicted: 15.8 per 100 (95%CI 11.7 to 20.0)), July 2009 and November 2011 were identified as critical time-points. | Yes |
| Yadav  (2004) | CEA vs. CAS | 747 | CUA | R | received external funding | CAS is non-inferior compared to CEA, however CAS is associated with higher costs. | Hussain (2016), Ontario, Canada | A decrease is seen in the rate of CEA (*p*=.06), while an increase is seen in the rate of CAS (*p*=.01) after 2004. | yes |
| 1. yes, no, unclear: according to authors impact paper 2. randomized (R), observational (O), meta-analysis (M) 3. not applicable 4. blind RCT | | | | | | | | | |
