# Supplementary material for: Impact of surgical intervention trials on healthcare: A systematic review of assessment methods, healthcare outcomes, and determinants
Source: PLoS One. 2020 May 22;15(5):e0233318. doi: 10.1371/journal.pone.0233318 (PMC7244162; doi:10.1371/journal.pone.0233318)
Supplement: S3 Table — (DOCX) [file pone.0233318.s004.docx]

**Table 8. Risk of Bias of Trial Papers**

| Paper | CSA | ICP | PCD | EAA | UASE | FU | LFU | PSZC | ACG | CG | BE | ASA | Total |
| --- | --- | --- | --- | --- | --- | --- | --- | --- | --- | --- | --- | --- | --- |
| Blakely | 2 | 2 | 2 | 2 | 2 | 2 | 2 | 2 | 2 | 2 | 1 | 2 | 23 |
| Blichert-Toft | 1 | 2 | 2 | 2 | 2 | 2 | 2 | 2 | 2 | 2 | 2 | 0 | 21 |
| Boughey | 2 | 2 | 2 | 2 | 0 | 2 | 0 | 2 | 0 | 2 | 0 | 0 | 14 |
| Brott | 2 | 2 | 2 | 2 | 2 | 1 | 2 | 2 | 2 | 2 | 2 | 2 | 23 |
| Bruin | 2 | 2 | 2 | 2 | 2 | 2 | 2 | 0 | 2 | 2 | 2 | 2 | 22 |
| Buchbinder | 2 | 2 | 2 | 2 | 2 | 2 | 2 | 2 | 2 | 2 | 2 | 2 | 24 |
| CSTS | 2 | 2 | n/a | 2 | 2 | n/a | 2 | n/a | 2 | 2 | 2 | 2 | 18 |
| Costa | 2 | 2 | 2 | 2 | 2 | 2 | 2 | 2 | 2 | 2 | 2 | 2 | 24 |
| ECSTC | 2 | 2 | 2 | 2 | 0 | 2 | 2 | 0 | 2 | 2 | 2 | 2 | 20 |
| EVAR | 2 | 2 | 2 | 2 | 0 | 1 | 2 | 2 | 2 | 2 | 2 | 2 | 21 |
| Fisher | 2 | 2 | 0 | 0 | 0 | 2 | 0 | 2 | 2 | 2 | 2 | 0 | 14 |
| Greenhalgh | 2 | 2 | 2 | 2 | 0 | 2 | 2 | 0 | 2 | 2 | 2 | 0 | 18 |
| Guiliano | 2 | 2 | 2 | 2 | 0 | 2 | 1 | 2 | 2 | 2 | 2 | 2 | 21 |
| Hofmeijer | 2 | 0 | 2 | 2 | 2 | 2 | 2 | 2 | 2 | 2 | 2 | 2 | 22 |
| Hughes | 2 | 2 | 2 | 2 | 0 | 2 | 0 | 2 | 2 | 2 | 2 | 2 | 20 |
| Juttler | 2 | 1 | 2 | 2 | 1 | 2 | 0 | 2 | 2 | 2 | 2 | 2 | 20 |
| Kallmes | 2 | 2 | 2 | 2 | 2 | 2 | 2 | 2 | 2 | 2 | 2 | 2 | 24 |
| Kirkley | 2 | 2 | 2 | 2 | 2 | 2 | 2 | 2 | 2 | 2 | 2 | 2 | 24 |
| Klazen | 2 | 2 | 2 | 2 | 0 | 2 | 2 | 2 | 2 | 2 | 2 | 2 | 22 |
| Kocher | 2 | 2 | 2 | 2 | 0 | 2 | 2 | 2 | 2 | 2 | 2 | 2 | 22 |
| Lederle | 2 | 2 | 2 | 2 | 2 | 2 | 2 | 2 | 2 | 2 | 2 | 2 | 24 |
| Liem | 2 | 2 | 2 | 2 | 2 | 2 | 2 | 2 | 2 | 2 | 2 | 2 | 24 |
| Mas | 2 | 2 | 2 | 2 | 2 | 2 | 2 | 2 | 2 | 2 | 2 | 2 | 24 |
| Mendelow | 2 | 2 | 2 | 2 | 0 | 2 | 1 | 2 | 2 | 2 | 2 | 2 | 21 |
| Molyneux | 2 | 2 | 2 | 2 | 2 | 2 | 2 | 2 | 2 | 2 | 2 | 2 | 24 |
| Moseley | 2 | 0 | 2 | 2 | 2 | 2 | 0 | 2 | 2 | 2 | 2 | 2 | 20 |
| NASCETC | 0 | 0 | 2 | 2 | 2 | 2 | 2 | 0 | 2 | 2 | 2 | 2 | 18 |
| Nelson | 2 | 2 | 2 | 2 | 2 | 2 | 2 | 0 | 2 | 2 | 2 | 2 | 22 |
| Patchell | 1 | 2 | 2 | 2 | 2 | 2 | 2 | 2 | 2 | 2 | 2 | 2 | 23 |
| Prinssen | 2 | 2 | 2 | 2 | 2 | 1 | 2 | 2 | 2 | 2 | 2 | 2 | 23 |
| Rousing | 2 | 2 | 0 | 2 | 0 | 2 | 2 | 1 | 2 | 2 | 2 | 2 | 19 |
| Rovers | 1 | 2 | 0 | 2 | 0 | 2 | 2 | 0 | 2 | 2 | 2 | 2 | 17 |
| SPACE | 2 | 2 | 2 | 2 | 0 | 2 | 2 | 2 | 2 | 2 | 2 | 2 | 22 |
| Vahedi | 2 | 2 | 2 | 2 | 2 | 2 | 2 | 2 | 2 | 2 | 2 | 2 | 24 |
| van Staaij | 2 | 2 | 0 | 2 | 0 | 2 | 1 | 2 | 2 | 2 | 2 | 2 | 19 |
| Veronesi | 1 | 2 | 0 | 2 | 0 | 2 | 2 | 0 | 2 | 2 | 2 | 0 | 15 |
| Wardlaw | 2 | 2 | 2 | 2 | 0 | 2 | 2 | 2 | 2 | 2 | 2 | 2 | 22 |
| Willits | 2 | 2 | 2 | 2 | 0 | 2 | 2 | 2 | 2 | 2 | 2 | 2 | 22 |
| Yadav | 2 | 0 | 2 | 2 | 0 | 2 | 2 | 1 | 2 | 2 | 2 | 2 | 19 |
| CSA: Clearly Stated Aim, ICP: Inclusion of Consecutive Patients, PCD: Prospective Collection of Data, EAA: Endpoint Appropriate to the Aim, UASE: Unbiased Assessment of Study Endpoints, FU: Follow-Up, LFU: Loss to Follow-Up, PSZC: Prospective Sample Size Calculation, ACG: an Adequate Control Group, CG: Contemporary Groups, BE: Baseline Equivalence, ASA: Adequate Statistical Analyses, 0 = not reported, 1 = reported but inadequate, 2 = reported and adequate | | | | | | | | | | | | | |
