# Supplementary material for: Impact of surgical intervention trials on healthcare: A systematic review of assessment methods, healthcare outcomes, and determinants
Source: PLoS One. 2020 May 22;15(5):e0233318. doi: 10.1371/journal.pone.0233318 (PMC7244162; doi:10.1371/journal.pone.0233318)
Supplement: S1 File — (DOCX) [file pone.0233318.s005.docx]

**Search strategy 10-3-2020**

Pubmed:

<http://www.ncbi.nlm.nih.gov/pubmed?otool=leiden>

**(**(("Surgical Procedures, Operative"[majr] OR "Specialties, Surgical"[majr] OR "Diseases Category/surgery"[majr] OR surg*[tw] OR neurosurg*[tw] OR "operative"[tw]) AND ("Comparative Effectiveness Research"[Mesh] OR "Comparative Effectiveness Research"[tw] OR "Clinical Studies as Topic"[Mesh] OR "Randomized Controlled Trials as Topic"[Mesh] OR (("Clinical trials"[ti] OR "Randomized Trials"[ti] OR "Randomised Trials"[ti] OR "Randomized Studies"[ti] OR "Randomised Studies"[ti] OR "Controlled Trials"[ti] OR "Clinical trial"[ti] OR "Randomized Trial"[ti] OR "Randomised Trial"[ti] OR "Randomized Trials"[ti] OR "Randomised Trials"[ti] OR "Controlled Trial"[ti]) NOT "Clinical Study"[Publication Type])) AND ("Health Impact Assessment"[Mesh] OR "impact"[tw] **OR "impacted"[tw] OR impact*[tw]** OR "payback"[tw] OR "return"[tw] OR "value"[tw] **OR "valued"[tw]** OR "Program Evaluation"[Mesh] OR "Evaluation Studies"[Publication Type] OR "Technology Assessment, Biomedical"[mesh] OR "technology assessment"[tw] OR "influence"[tw] **OR influenc*[tw]** OR "consequence"[tw] **OR "consequences"[tw]** OR "importance"[tw] OR "burden"[tw] **OR "burdens"[tw]** OR "budget impact analysis"[tw] **OR "budget impact"[tw]**) AND ("Professional Practice"[Mesh] OR "practice"[tw] OR "clinical practice"**[tw]** OR **"pattern"[tw]** OR "patterns"[tw] OR "Practice Patterns, Physicians'"[Mesh] OR "Delivery of Health Care"[Mesh] OR "Evidence-Based Practice"[Mesh] OR "Evidence-Based"[tw] OR "Health Care Costs"[Mesh] OR "Costs"[tw] OR **"Cost"[tw] OR "money"[tw] OR "budget"[tw] OR budget*[tw]** OR "trends"[tw] **OR "trend"[tw]** OR **"attitude"[tw] OR "attitudes"[tw] OR "policy"[tw] OR "policies"[tw]**) **NOT (("case reports"[ptyp] OR "case report"[ti] OR "review"[ptyp] OR "review"[ti]) NOT "clinical study"[ptyp])) OR** (("Surgical Procedures, Operative"[majr] OR "Specialties, Surgical"[majr] OR "Diseases Category/surgery"[majr] OR surg*[ti] OR neurosurg*[ti] OR "operative"[ti]) AND ("Comparative Effectiveness Research"[Mesh] OR "Comparative Effectiveness Research"[tw] OR "Clinical Studies as Topic"[Mesh] OR "Randomized Controlled Trials as Topic"[Mesh] OR "Clinical trials"[ti] OR "Randomized Trials"[ti] OR "Randomised Trials"[ti] OR "Randomized Studies"[ti] OR "Randomised Studies"[ti] OR "Controlled Trials"[ti] OR "Clinical trial"[ti] OR "Randomized Trial"[ti] OR "Randomised Trial"[ti] OR "Randomized Trials"[ti] OR "Randomised Trials"[ti] OR "Controlled Trial"[ti]) AND ("Health Impact Assessment"[majr] OR "impact"[ti] **OR "impacted"[ti] OR impact*[ti]** OR "payback"[ti] OR "return"[ti] OR "value"[ti] **OR "valued"[ti]** OR "Program Evaluation"[majr] OR "Evaluation Studies"[Publication Type] OR "Technology Assessment, Biomedical"[majr] OR "technology assessment"[ti] OR "influence"[ti] **OR influenc*[ti]** OR "consequence"[ti] **OR "consequences"[ti]** OR "importance"[ti] OR "burden"[ti] **OR "burdens"[ti]** OR "budget impact analysis"[ti] **OR "budget impact"[ti]**) AND ("Professional Practice"[Mesh] OR "practice"[tw] OR "clinical practice"**[tw]** OR **"pattern"[tw]** OR "patterns"[tw] OR "Practice Patterns, Physicians'"[Mesh] OR "Delivery of Health Care"[Mesh] OR "Evidence-Based Practice"[Mesh] OR "Evidence-Based"[tw] OR "Health Care Costs"[Mesh] OR "Costs"[tw] OR **"Cost"[tw] OR "money"[tw] OR "budget"[tw] OR budget*[tw]** OR "trends"[tw] **OR "trend"[tw]** OR **"attitude"[tw] OR "attitudes"[tw] OR "policy"[tw] OR "policies"[tw]**) **NOT (("case reports"[ptyp] OR "case report"[ti] OR "review"[ptyp] OR "review"[ti]) NOT "clinical study"[ptyp])))**

MEDLINE:

<http://gateway.ovid.com/ovidweb.cgi?T=JS&MODE=ovid&NEWS=n&PAGE=main&D=prmz>

Embase:

<http://ovidsp.ovid.com/ovidweb.cgi?T=JS&PAGE=main&MODE=ovid&D=oemezd>

(**(exp *"surgery"/ OR** surg*.ti,ab OR neurosurg*.ti,ab OR "operative".ti,ab**) AND (exp "randomized controlled trial (topic)" OR "clinical trial (topic)"/ OR exp "comparative effectiveness"/** **OR "**Clinical trials".ti OR "Randomized Trials".ti OR "Randomised Trials".ti OR "Randomized Studies".ti OR "Randomised Studies".ti OR "Controlled Trials".ti OR "Randomized Trials".ti OR "Randomised Trials".ti OR (("Clinical trial".ti OR "Randomized Controlled Trial".ti **OR "**Clinical trials".ti OR "Randomized Trials".ti OR "Randomised Trials".ti OR "Randomized Studies".ti OR "Randomised Studies".ti OR "Controlled Trials".ti OR "Clinical trial".ti OR "Randomized Trial".ti OR "Randomised Trial".ti OR "Randomized Trials".ti OR "Randomised Trials".ti OR "Controlled Trial".ti) NOT exp "Clinical Trial"/)) AND ("impact".ti,ab OR impact*.ti,ab OR "payback".ti,ab OR "return".ti,ab OR *"value"/ OR "influence".ti,ab OR exp "biomedical technology assessment"/ OR "biomedical technology assessment".ti,ab OR exp "program evaluation"/ OR exp "evaluation study"/ OR "value".ti,ab OR "valued".ti,ab OR "clinical practice".ti,ab OR "influence".ti,ab OR influenc*.ti,ab OR "consequence".ti,ab OR "consequences".ti,ab OR "importance".ti,ab OR "burden".ti,ab OR "burdens".ti,ab OR "budget impact analysis".ti,ab OR "budget impact".ti,ab) AND (exp *"Professional Practice"/ OR "clinical practice"/ OR "practice".ti,ab OR "clinical practice".ti,ab OR "pattern".ti,ab OR "patterns".ti,ab OR exp *"Health Care Delivery"/ OR exp *"Evidence-Based Practice"/ OR "Evidence-Based".ti,ab OR exp *"Health Care Cost"/ OR "Costs".ti,ab OR "Cost".ti,ab OR "money".ti,ab OR "budget".ti,ab OR budget*.ti,ab OR "trends".ti,ab OR "trend".ti,ab OR "attitude".ti,ab OR "attitudes".ti,ab OR "policy".ti,ab OR "policies".ti,ab) **NOT ((exp "case report"/ OR "case report".ti OR "review"/ OR "review".ti) NOT ("clinical study"/ OR exp "clinical trial"/))** NOT (conference review or conference abstract).pt)

Web of Science

<http://isiknowledge.com/wos>

(ts=**(**surg* OR neurosurg* OR "operative"**) AND ti=("**Clinical trials" OR "Randomized Trials" OR "Randomised Trials" OR "Randomized Studies" OR "Randomised Studies" OR "Controlled Trials" OR "Randomized Trials" OR "Randomised Trials") AND ts=("impact" OR impact* OR "payback" OR "return" OR "value" OR "influence" OR "biomedical technology assessment" OR "biomedical technology assessment" OR "program evaluation" OR "evaluation study" OR "value" OR "valued" OR "clinical practice" OR "influence" OR influenc* OR "consequence" OR "consequences" OR "importance" OR "burden" OR "burdens" OR "budget impact analysis" OR "budget impact") AND ts=("Professional Practice" OR "clinical practice" OR "practice" OR "clinical practice" OR "pattern" OR "patterns" OR "Health Care Delivery" OR "Evidence-Based Practice" OR "Evidence-Based" OR "Health Care Cost" OR "Costs" OR "Cost" OR "money" OR "budget" OR budget* OR "trends" OR "trend" OR "attitude" OR "attitudes" OR "policy" OR "policies") **NOT ti=(("case report" OR "case report" OR "review" OR "review") NOT ("clinical study" OR "clinical trial"))) OR** (ti=**(**surg* OR neurosurg* OR "operative"**) AND ts=("**Clinical trials" OR "Randomized Trials" OR "Randomised Trials" OR "Randomized Studies" OR "Randomised Studies" OR "Controlled Trials" OR "Randomized Trials" OR "Randomised Trials") AND ts=("impact" OR impact* OR "payback" OR "return" OR "value" OR "influence" OR "biomedical technology assessment" OR "biomedical technology assessment" OR "program evaluation" OR "evaluation study" OR "value" OR "valued" OR "clinical practice" OR "influence" OR influenc* OR "consequence" OR "consequences" OR "importance" OR "burden" OR "burdens" OR "budget impact analysis" OR "budget impact") AND ts=("Professional Practice" OR "clinical practice" OR "practice" OR "clinical practice" OR "pattern" OR "patterns" OR "Health Care Delivery" OR "Evidence-Based Practice" OR "Evidence-Based" OR "Health Care Cost" OR "Costs" OR "Cost" OR "money" OR "budget" OR budget* OR "trends" OR "trend" OR "attitude" OR "attitudes" OR "policy" OR "policies") **NOT ti=(("case report" OR "review") NOT ("clinical study" OR "clinical trial")))** NOT conference abstract **NOT ti=(veterinary OR rabbit OR rabbits OR animal OR animals OR mouse OR mice OR rodent OR rodents OR rat OR rats OR pig OR pigs OR porcine OR horse* OR equine OR cow OR cows OR bovine OR goat OR goats OR sheep OR ovine OR canine OR dog OR dogs OR feline OR cat OR cats))**

**Cochrane**

<http://www.cochranelibrary.com/>

(**(**surg* OR neurosurg* OR "operative"**) AND ("**Clinical trials" OR "Randomized Trials" OR "Randomised Trials" OR "Randomized Studies" OR "Randomised Studies" OR "Controlled Trials" OR "Randomized Trials" OR "Randomised Trials") AND ("impact" OR impact* OR "payback" OR "return" OR "value" OR "influence" OR "biomedical technology assessment" OR "biomedical technology assessment" OR "program evaluation" OR "evaluation study" OR "value" OR "valued" OR "clinical practice" OR "influence" OR influenc* OR "consequence" OR "consequences" OR "importance" OR "burden" OR "burdens" OR "budget impact analysis" OR "budget impact") AND ("Professional Practice" OR "clinical practice" OR "practice" OR "clinical practice" OR "pattern" OR "patterns" OR "Health Care Delivery" OR "Evidence-Based Practice" OR "Evidence-Based" OR "Health Care Cost" OR "Costs" OR "Cost" OR "money" OR "budget" OR budget* OR "trends" OR "trend" OR "attitude" OR "attitudes" OR "policy" OR "policies")**):ti,ab,kw**
